# Therapeutic Potential of Crocin and Nobiletin in a Mouse Model of Dry Eye Disease: Modulation of the Inflammatory Response and Protection of the Ocular Surface

**DOI:** 10.5812/ijpr-149463

**Published:** 2024-09-15

**Authors:** Ahmad Habibian Sezavar, Seyed Nasser Ostad, Yazdan Hasani Nourian, Hossein Aghamollaei

**Affiliations:** 1 Department of Toxicology and Pharmacology, Faculty of Pharmacy, Tehran University of Medical Sciences (TUMS), Tehran, Iran; 2 Chemical Injuries Research Center, Systems Biology and Poisonings Institute, Baqiyatallah University of Medical Sciences, Tehran, Iran

**Keywords:** Dry Eye Disease, Inflammation, Crocin, Nobiletin, Lacrimal Gland Excision

## Abstract

**Background:**

Dry eye disease (DED) is a multifactorial condition characterized by ocular surface inflammation, tear film instability, and corneal epithelial damage. Current treatments often provide temporary relief without addressing the underlying inflammatory mechanisms.

**Objectives:**

This study examined the therapeutic potential of crocin and nobiletin, two naturally derived compounds with well-known antioxidant and anti-inflammatory properties, in a mouse model of DED induced by lacrimal gland excision (LGE).

**Methods:**

Thirty female Balb/c mice were divided into five groups (n = 6 each): Control (sham surgery), untreated DED, nobiletin-treated DED (32.75 µM), crocin-treated DED (34 µM), and 1% betamethasone-treated DED. Treatments were administered three times daily for 28 days. Ocular tissues were evaluated using Hematoxylin and Eosin (H&E) staining and fluorescein staining. Conjunctival inflammatory cytokines, including interleukin-6 (IL-6), interleukin-1 beta (IL-1β), and tumor necrosis factor-alpha (TNF-α), were measured by enzyme-linked immunosorbent assay (ELISA).

**Results:**

Histological analysis showed that the crocin and nobiletin treatment groups exhibited reduced epithelial disruption, keratinization, and inflammatory cell infiltration compared to the untreated DED group. The ELISA assay revealed that both compounds efficiently inhibited the production of the pro-inflammatory cytokines IL-6, TNF-α, and IL-1β, which are key mediators of DED pathogenesis. Fluorescein staining further confirmed the protective impact of crocin and nobiletin on corneal epithelial integrity. Moreover, the anti-inflammatory and epithelial-preserving effects of these compounds were comparable to those of the corticosteroid betamethasone.

**Conclusions:**

Overall, these findings suggest that crocin and nobiletin have therapeutic potential for DED management by modulating inflammatory responses and enhancing ocular surface healing. These naturally derived compounds offer promising avenues for the development of safer and more effective treatments for this challenging condition. However, further investigations, including clinical trials, are essential to elucidate the underlying mechanisms of action and optimize therapeutic approaches.

## 1. Background

Dry eye disease (DED) is a widespread and concerning ocular condition, affecting a quarter of individuals who visit ophthalmic clinics, making it an increasingly significant public health issue (1). Dry eye disease leads to tear film instability, discomfort, and visual disturbances, which can result in damage and ulceration of the cornea (2, 3). The consequences of untreated or chronic DED can be severe, underscoring the importance for eye care practitioners to effectively detect and manage this disease to reduce patient suffering and prevent long-term ocular complications (4).

Inflammation is the primary mechanism underlying DED. During inflammation, there is an infiltration of immune cells and pro-inflammatory cytokines such as interleukin 6 (IL-6), interleukin 1 (IL-1), tumor necrosis factor-alpha (TNF-α), and leukocytes (5, 6). Environmental stressors, such as exposure to ultraviolet light, can trigger the activation of various phosphatase enzymes and ocular kinases, including the mitogen-activated protein (MAP) kinase signaling cascade (7). This inflammatory environment and the abnormal regulation of enzymes that remodel the extracellular matrix can eventually lead to damage to the delicate eye surface, contributing to the onset and progression of DED (5, 8).

Crocin, a water-soluble bioactive component derived from saffron, has garnered increasing scientific interest due to its diverse pharmacological properties (9). Existing research has demonstrated that crocin may exert protective effects against retinal stress, in addition to its well-documented anti-inflammatory and antioxidant properties (10). Crocin also exhibits potent immunomodulatory effects through various mechanisms, including the reduction of matrix metalloproteinases (MMP-3 and MMP-9), the pro-inflammatory cytokines TNF-α and IL-6, and the chemokine monocyte chemoattractant protein-1 (MCP-1) in the serum (11). These multifaceted biological activities of crocin underscore its potential therapeutic applications in the management of ocular and inflammatory disorders (11, 12).

Nobiletin, a unique polymethoxylated flavonoid, is abundantly present in the peels of citrus fruits. This group of polymethoxyflavones (PMFs) has been extensively studied, with animal research demonstrating their potential health-promoting effects (13). Nobiletin has been investigated for its anti-tumor, anticonvulsive, and anti-inflammatory properties, and has also been studied for its potential applications in the treatment of cognitive deficits (14). Research has shown that nobiletin can reduce the expression of genes responsible for the production of pro-inflammatory cytokines, including IL-1α, IL-1β, TNF-α, and IL-6 (14).

## 2. Objectives

Given that inflammation is a hallmark of DED, and previous research has confirmed the effective anti-inflammatory properties of nobiletin and crocin, this study aimed to evaluate the efficacy of these two compounds in a mouse model of DED.

## 3. Methods

### 3.1. Animals

Thirty adult male Balb/c mice, weighing approximately 22 ± 2 grams and aged 8 weeks, were obtained from the Animal Center at the Royan Institute in Iran. The animals were maintained in a controlled environment, adhering to optimal standards regarding light/dark cycles, humidity levels, and ambient temperature. They were provided with ad libitum access to standard dietary provisions and potable water.

### 3.2. Cell Culture

The HEK-293 cell line was acquired from the National Cell Bank of Iran, situated at the Pasteur Institute in Tehran. These cells were propagated in Roswell Park Memorial Institute (RPMI) medium, supplemented with 10% fetal bovine serum (FBS), 100 units/mL penicillin, and 100 μg/mL streptomycin. The culture conditions maintained were a humidified incubator set at 37°C, with 5% CO_2_ and 95% humid air. The culture medium was replenished twice per week until the cells reached 80 - 90% confluency. Subsequently, the cells were dissociated using trypsin and subcultured for passaging and cryopreservation. All experiments were conducted using cells within the passage number range of 3 - 6. Cells were routinely tested for mycoplasma contamination using Hoechst staining. Briefly, cells were fixed and stained with Hoechst 33258 dye, which binds to DNA. Samples were then examined under a fluorescence microscope for the presence of extranuclear DNA, indicative of mycoplasma infection. All cell cultures used in this study were confirmed to be mycoplasma-negative.

### 3.3. Cell Proliferation Assay

The MTT (3-(4,5-dimethylthiazolyl)-2,5-diphenyl-tetrazolium bromide) assay was performed to evaluate the individual effects of crocin and nobiletin on cell survival. In this study, (1 × 10^4^) Hek-293 cells were seeded per well in 96-well plates. After a 24-hour incubation period, the cell culture medium was removed, and the cells were exposed to varying concentrations of nobiletin (12.5, 25, 50, 100, 200, 400 μM) and crocin (12.5, 25, 50, 100, 200, 400 μM) for 48 hours. The measurement of absorbance was performed at an optical density (OD) of 570 nm. Cell viability was assessed by comparing the absorbance values of the drug-treated cells to those of untreated control cells. The obtained data were then plotted as a percentage of the control, representing relative cell viability. The concentration ranges for crocin and nobiletin were selected based on previous studies reported in the literature (15-17). To encompass the reported effective ranges and explore potential dose-dependent effects, a concentration range was chosen that allows for the evaluation of cellular responses at concentrations below, near, and above the previously reported half-maximal inhibitory concentrations (IC_50_) values. This approach provides a comprehensive assessment of the compounds' effects on Hek-293 cells while building upon the findings of earlier research in the field.

### 3.4. Safety of Substances in Rabbit Eye

To assess the ocular toxicity of the two compounds, crocin and nobiletin, an in vivo eye irritation test was conducted. The concentrations evaluated were strategically chosen based on the respective IC_50_ of each compound. Specifically, the concentrations tested included the IC_50_ value itself, as well as concentrations at one-half and one-quarter of the IC_50_ value. This approach allowed for a comprehensive evaluation of the potential eye irritation caused by varying levels of exposure to these compounds. The experimental protocol involved the preparation of stock solutions for crocin and nobiletin using DMSO as the solvent at high concentrations. These stock solutions were then diluted with an artificial tear solution to obtain the desired treatment doses for instillation into the eye conjunctiva of the test subjects. Subsequently, the team meticulously observed and recorded any occurrences of eye symptoms, focusing on the iris, cornea, and conjunctiva over periods of 1, 24, 48, and 72 hours (18).

For crocin, the doses tested were 272, 136 (IC_50_), 68, and 34 μM. For nobiletin, the doses tested were 262, 131 (IC_50_), 65.5, and 32.75 μM.

### 3.5. Dry Eye Disease Induction by Lacrimal Gland Excision and Treatment

The mouse model of DED induction via lacrimal gland excision (LGE) has been detailed in our previous study (19). The animals were randomly divided into five groups of six mice each: (1) control group, mice underwent a sham surgery involving a 5-mm incision without LGE, followed by ocular instillation of 3 microliters of artificial tears three times daily for 28 days; (2) LGE group, mice underwent LGE, followed by instillation of 3 microliters of artificial tears three times daily for 28 days; (3) nobiletin group, mice underwent LGE, followed by ocular instillation of 3 microliters of 32.75 µM nobiletin three times daily for 28 days; (4) crocin group, mice underwent LGE excision, followed by ocular instillation of 3 microliters of 34 µM crocin three times daily for 28 days; (5) BET group, mice underwent LGE excision, followed by ocular instillation of 3 microliters of 1% betamethasone eye drops three times daily for 28 days.

### 3.6. Fluorescein Staining

To evaluate the extent of corneal damage following the excision of the extraorbital lacrimal gland (ELG) and the subsequent induction of dry eye, corneal fluorescein staining was performed at specific time points: On the day of the procedure (day 0), as well as 14 days and 28 days after the surgical intervention. In brief, a fluorescein stripe was applied to the eyes under anesthesia. An ocular examination was then conducted using a microscope equipped with a blue light filter to facilitate visualization. Images were captured using a digital camera, and the extent of corneal epithelial damage was quantified using a grading system, wherein higher fluorescein staining intensities corresponded to greater epithelial disruption. The evaluation of corneal staining was conducted based on a scoring system: A score of 0 indicated no detectable staining; a score of 0.5 corresponded to minimal punctate staining; a score of 1 was assigned to diffuse punctate staining; a score of 2 represented diffuse staining affecting up to one-third of the corneal surface; diffuse staining involving more than one-third of the cornea was given a score of 3; a score of 4 was attributed to staining encompassing over two-thirds of the corneal area (20).

### 3.7. Hematoxylin and Eosin Staining

Following the euthanasia procedure, the entire right-eye globes of the mice were collected. The ocular specimens were immersed in a 10% formalin solution for one week to facilitate fixation. After fixation, the tissues were embedded in paraffin, and 3 μm thick sections were obtained using a microtome. These tissue sections were then subjected to hematoxylin and eosin (H&E) staining to allow for the evaluation of pathological alterations under microscopic examination.

### 3.8. Cytokine Analysis

For the quantification of pro-inflammatory cytokine levels, ocular specimens from half of the animals in each experimental group were harvested and stored at -80°C. The concentrations of IL-1β, TNF-α, and IL-6 were determined using enzyme-linked immunosorbent assay (ELISA) kits. Eye tissue homogenates were prepared by adding normal saline solution to the whole eye specimens at a ratio of 10% weight/volume. The homogenized samples were then centrifuged at 12,000 revolutions per minute (RPM) for 30 minutes at 4°C, and the supernatant fractions were collected. The levels of IL-1β, TNF-α, and IL-6 in the eye homogenate supernatants were subsequently measured using commercially available ELISA kits (Carmania Pars Gene, Tehran, Iran) following the manufacturer's recommended protocols.

### 3.9. Statistical Examination

Statistical analyses were conducted using GraphPad Prism 8.0 software. The data are presented as mean ± standard error of the mean (SEM). To evaluate statistical significance, one-way and two-way analysis of variance (ANOVA) were performed, followed by Tukey's post hoc test for multiple comparisons. A P-value of less than 0.05 was considered statistically significant.

## 4. Results

### 4.1. MTT Assay

**Figure 1.**
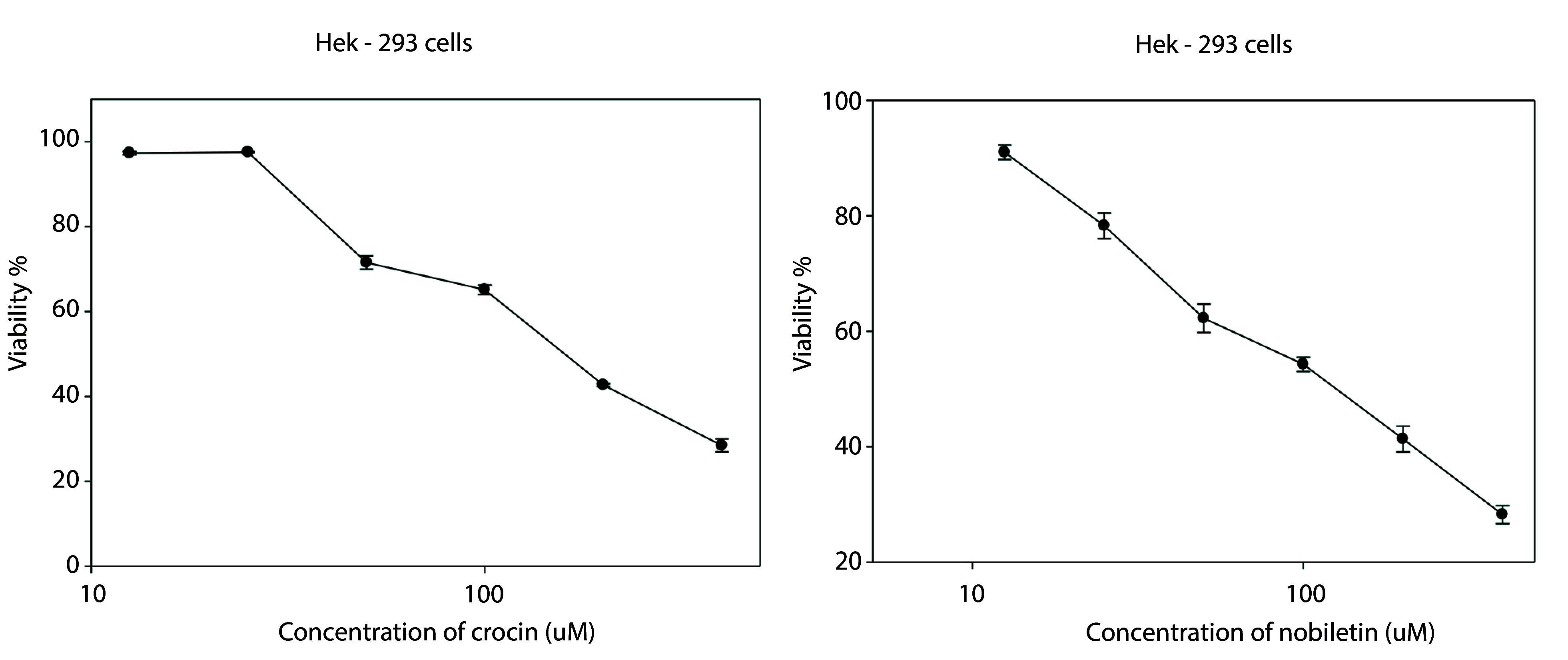
MTT cell viability assay of HEK-293 cells following 48-hour treatment with crocin (400, 200, 100, 50, 25, and 12.5 μM) and nobilitin (400, 200, 100, 50, 25, and 12.5 μM)

Viability findings showed that both nobiletin and crocin exhibited a concentration-dependent cytotoxic effect on HEK-293 cells. IC_50_ for cell viability were determined to be 136 μM for crocin and 131 μM for nobiletin (Figure 1 and Table 1). 

**Table 1.** The IC_50_ Value of Crocin (µM) and Nobiletin (µM) on HEK-293 Cell

Cell line	IC_50_ µM	
	Crocin	Nobiletin
**HEK-293**	136	131

### 4.2. Safety of Substances in the Rabbit Eye

**Figure 2.**
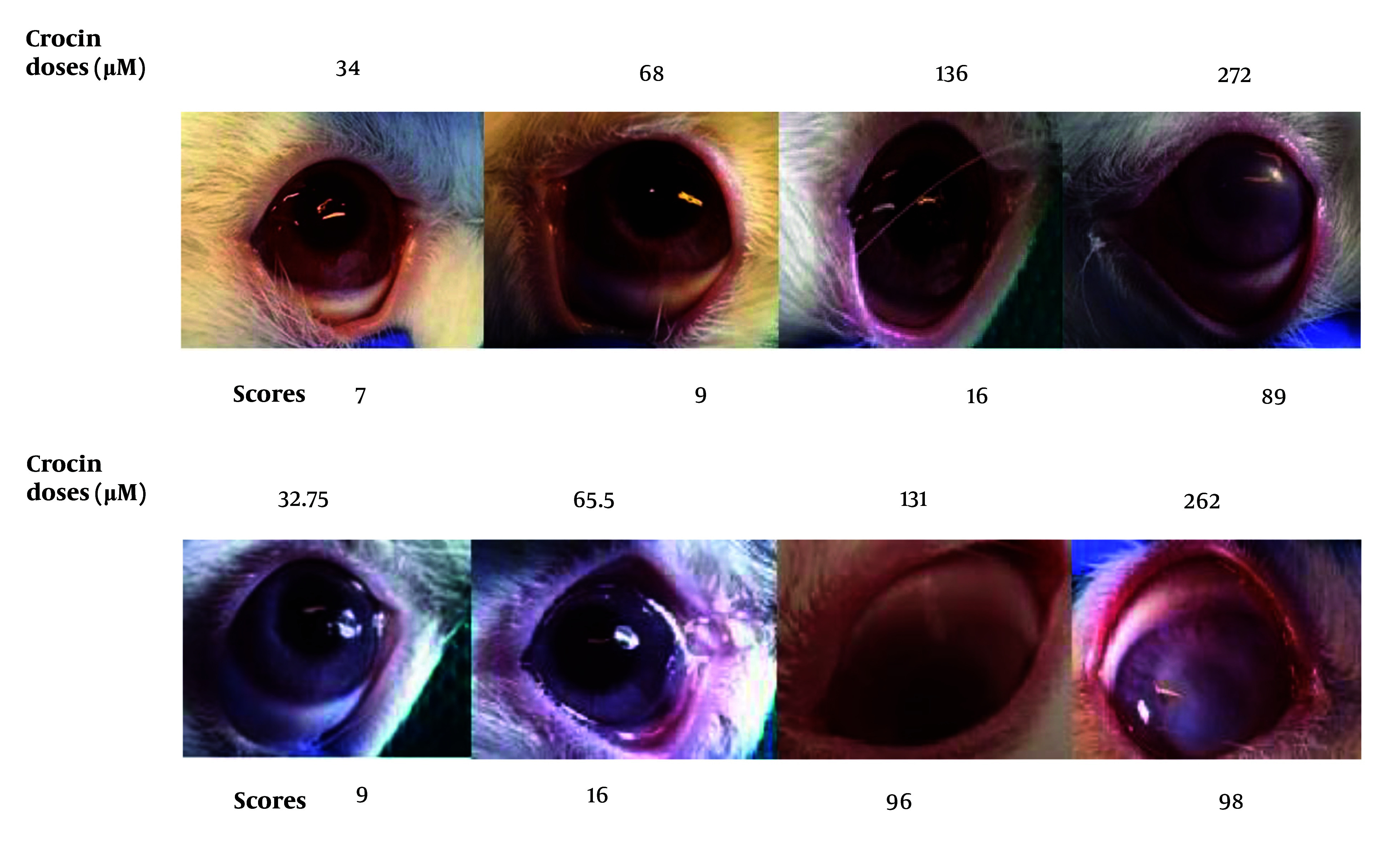
Eye Irritation Test for crocin and nobiletin based on the Draize test in rabbits' eyes. Single drop application, followed by evaluation at 1, 24, 48, and 72-hour time points.

Based on the safety assay results on rabbit eyes, doses of 34 μM for crocin and 32.75 μM for nobiletin were selected for further evaluation in animal studies. At elevated concentrations of nobiletin and crocin, ocular turbidity was observed, along with mild conjunctival hyperemia (Figure 2 and Table 2). 

**Table 2.** Eye Irritation Test for Crocin and Nobiletin in Rabbits' Eyes

Variables	Values			
**Crocin Doses (µM)**	34 ^a^	68	136	272
**Scores**	7	9	16	89
**Nobiletin Doses (µM)**	32.75 ^a^	65.5	131	262
**Scores**	9	16	96	98

^a^ Indicate the highest safe concentration of each compound.

### 4.3. Quantification of TNF-α Concentrations in LPS-Stimulated HEK-293 Cell Line

**Figure 3.**
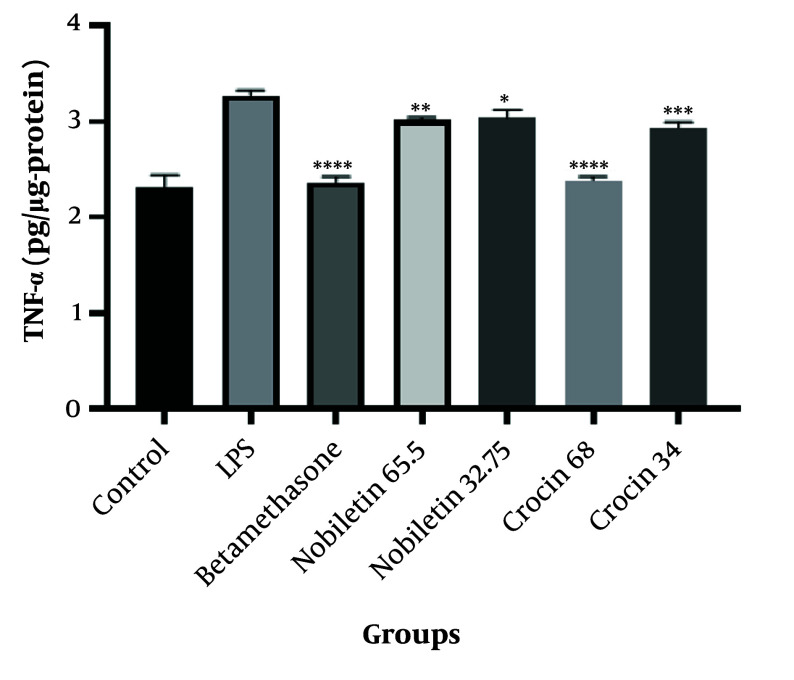
Tumor necrosis factor-alpha (TNF-α) concentration in HEK-293 cells treated with LPS (1 μg/mL) for 48 hours. Control, LPS, betamethasone, crocin (34 μM and 68 μM), and nobiletin (32.75 μM and 65.5 μM) treatment groups are shown. * P < 0.05, ** P < 0.01, *** P < 0.001, **** P < 0.0001 compared to the LPS group.

The results demonstrated that both crocin and nobiletin exhibited anti-inflammatory properties at all tested concentrations. However, the higher doses of crocin (68 μM) and nobiletin (65.5 μM) showed superior anti-inflammatory efficacy compared to their lower-dose counterparts (Figure 3). 

### 4.4. Crocin and Nobiletin Improved Corneal Damage

**Figure 4.**
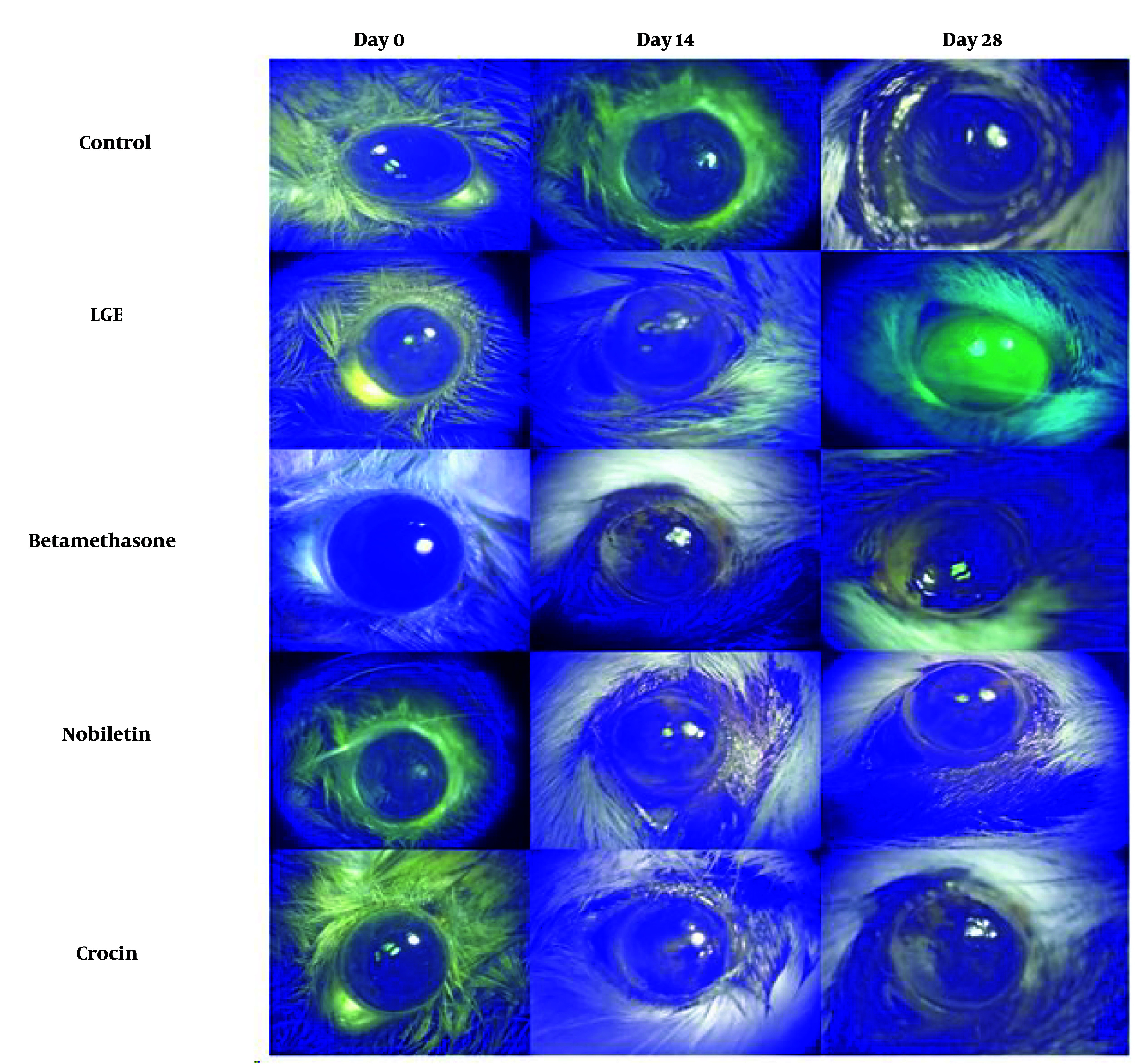
Assessment of corneal epithelial defects using fluorescein dye staining at 0, 14 and 28-day intervals following lacrimal gland excision (LGE)

**Figure 5.**
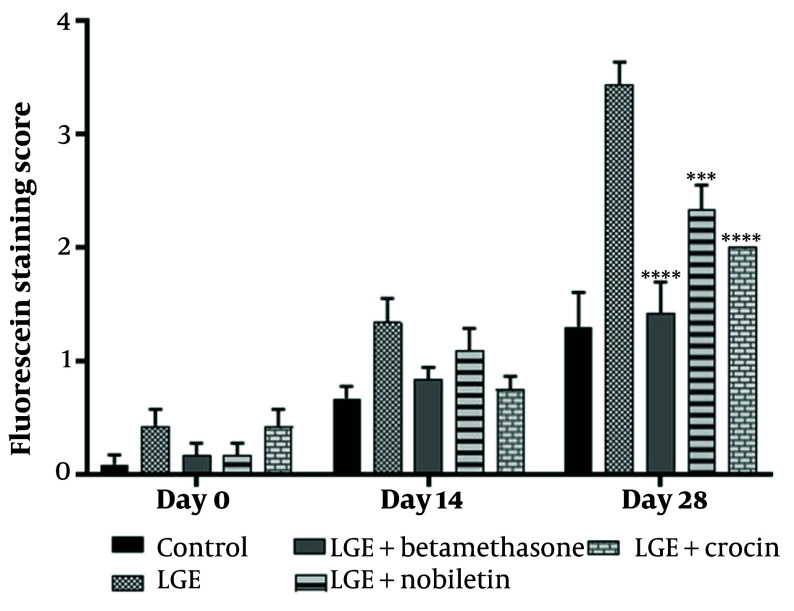
Quantitative analysis of fluorescein staining scores at 0, 14, and 28 days following lacrimal gland excision (LGE). *** P < 0.001, **** P < 0.0001

The results demonstrated that the betamethasone positive control group, as well as the crocin and nobiletin treatment groups, exhibited a significant reduction in corneal epithelial damage compared to the LGE group. There was no significant difference in the extent of corneal epithelial damage between the crocin and nobiletin groups and the betamethasone group (Figures 4 and 5). 

### 4.5. Hematoxylin and Eosin Staining

In the LGE group, severe angiogenesis and edema were evident, along with significant infiltration of inflammatory cells. These findings indicate a severe inflammatory condition and tissue damage in this group, consistent with the pathophysiology of DED. In contrast, the group treated with crocin exhibited angiogenesis, epithelial damage, edema, and mild disorganization in collagen, but no infiltration of inflammatory cells was noted. These results suggest that crocin effectively prevented tissue damage and inflammation, although some epithelial damage and edema persisted.

In the group treated with nobiletin, moderate levels of angiogenesis, epithelial damage, edema, and collagen disorder were observed, along with the infiltration of inflammatory cells. This indicates that nobiletin was able to partially prevent tissue damage and inflammation, but its effects were less pronounced compared to crocin. However, both natural compounds were able to partially prevent the progression of the disease.

**Figure 6.**
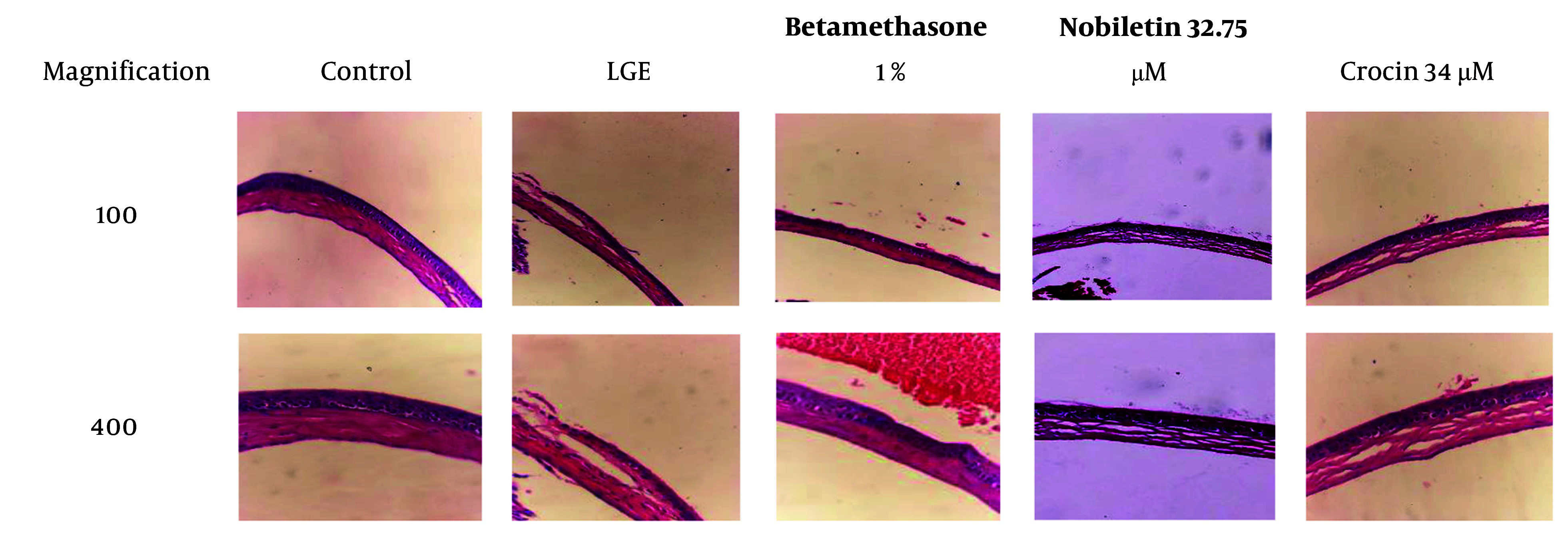
Histological evaluation of corneal tissue at 28 days post-operative following exorbital lacrimal gland excision (H&E staining, 100x and 400x)

In the betamethasone-treated group (positive control group), the levels of edema, angiogenesis, and epithelial damage were lower than in the groups treated with crocin and nobiletin. This finding demonstrates that betamethasone, as a potent corticosteroid, was able to significantly reduce inflammation and tissue damage. This result was expected, as betamethasone is a strong anti-inflammatory drug commonly used in the treatment of inflammatory conditions such as dry eye (Figure 6). 

### 4.6. Effect of Crocin and Nobiletin on Concentrations of IL-6, TNF-α, and IL-1β

The results indicated that for TNF-α, the treatment groups receiving crocin and nobiletin did not exhibit any statistically significant reduction compared to the LGE group. However, for the cytokines IL-6 and IL-1β, the treatment groups administered with crocin and nobiletin demonstrated a significant decrease in their levels compared to the LGE group. These findings suggest that both natural compounds, crocin and nobiletin, have the ability to mitigate the overproduction of these key pro-inflammatory cytokines, which are known to play a crucial role in the pathogenesis of DED.

Specifically, in the case of IL-6, the administration of crocin resulted in a statistically significant reduction in the concentration of this cytokine when compared to the LGE group (P < 0.0001). This finding demonstrates that crocin has strong anti-inflammatory effects in inhibiting IL-6 production. On the other hand, nobiletin showed a weaker result compared to crocin in relation to the LGE group (P < 0.001), although it still significantly reduced IL-6 levels.

**Figure 7.**
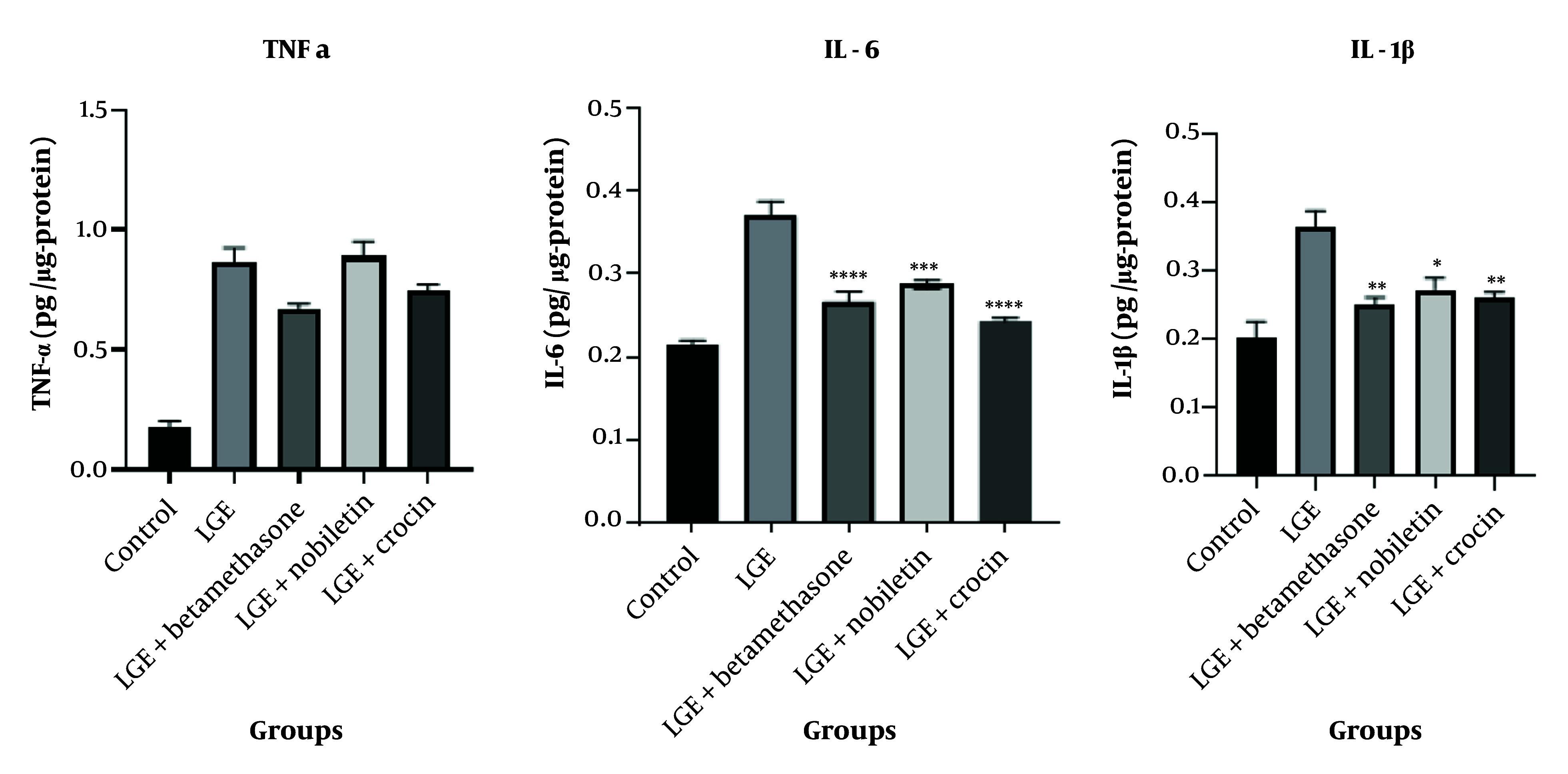
Assessment of conjunctival concentrations of pro-inflammatory cytokines: IL-6, IL-1β, and tumor necrosis factor-alpha (TNF-α). * P < 0.05, ** P < 0.01, *** P < 0.001, **** P < 0.0001 comparative to the LGE group

Regarding IL-1β, both the crocin and nobiletin treatment groups were able to significantly reduce the level of this cytokine compared to the LGE group. This suggests that both natural compounds possess anti-inflammatory properties in inhibiting the production of IL-1β, which is one of the key cytokines involved in the inflammation and pathogenesis of DED (Figure 7). 

## 5. Discussion

Dry eye disease is a multifactorial condition characterized by ocular surface inflammation, tear film instability, and corneal epithelial damage. This chronic condition can significantly impact the patient's quality of life, leading to eye discomfort, visual disturbances, and an increased risk of ocular surface complications (21). In this study, we investigated the therapeutic potential of crocin and nobiletin, two natural compounds with documented anti-inflammatory and antioxidant properties, in alleviating the symptoms and pathological changes associated with DED using a mouse model.

Histological analysis using H&E staining revealed significant epithelial disruption, keratinization, and inflammatory cell infiltration in the untreated DED group, consistent with the pathological features of DED. These observations align with previous studies that have documented ocular surface changes, including epithelial defects, goblet cell loss, and inflammatory cell infiltration in DED patients and animal models (5, 22). The betamethasone-treated group, serving as a positive control, showed reduced inflammatory changes and preserved epithelial integrity, reaffirming the well-established anti-inflammatory effect of corticosteroid therapy in DED management. Notably, the groups treated with crocin and nobiletin exhibited similar protective effects, with minimal epithelial disruption and reduced inflammatory cell infiltration. These findings suggest that nobiletin and crocin possess anti-inflammatory and epithelial-protective properties, which may contribute to their therapeutic potential in DED. The ability of these compounds to modulate the inflammatory response and promote epithelial integrity is particularly important, as chronic inflammation and epithelial damage are key factors in the pathogenesis and persistence of DED.

Several studies have demonstrated that crocin can attenuate the production of pro-inflammatory cytokines, including IL-6, TNF-α, and IL-1β, through the inhibition of the NF-κB and MAPK signaling pathways (17, 23). It may also downregulate inflammatory enzymes like COX-2 and iNOS (24). These combined anti-inflammatory and antioxidant effects of crocin could help control inflammation and oxidative damage in DED. Previous studies using crocin for treating experimental dry eye in rats and rabbits have demonstrated reduced inflammatory cell infiltration in the cornea and conjunctiva (19, 25).

The results of the ELISA assay further strengthened the anti-inflammatory effects of nobiletin and crocin. Elevated levels of pro-inflammatory cytokines, such as IL-6, IL-1β, and TNF-α, were observed in the untreated DED group, consistent with the inflammatory nature of the disease. The betamethasone group significantly reduced cytokine levels, further confirming its anti-inflammatory action. Our findings suggest that while these two natural compounds did not significantly reduce TNF-α levels, their mechanism of action is likely focused on other inflammatory pathways besides the TNF-α production pathway. The results obtained in our study are consistent with several previous investigations that have documented the anti-inflammatory features of crocin, which are mediated through the attenuation of specific pro-inflammatory cytokines, such as IL-6 and IL-1β, while no significant effect was observed on TNF-α levels (26).

The literature highlights the crucial involvement of IL-1 and IL-6 in driving the inflammatory processes underlying DED, with increased levels of these cytokines consistently observed in the tear film of patients. Notably, Solomon et al. reported an increase in the pro-inflammatory forms of IL-1 (IL-1α and mature IL-1β) and a decrease in the biologically inactive precursor IL-1β in dry eye patients, underscoring the importance of this cytokine in the disease pathology (23). Xiao et al. demonstrated that crocin treatment in a mouse model of depression effectively suppressed neuroinflammation and oxidative stress in the hippocampus, as evidenced by the attenuation of IL-1β levels (27). Similarly, our results showed that crocin effectively reduced inflammation in the DED model, as indicated by the decreased infiltration of inflammatory cells and minimal epithelial disruption. However, in contrast to its effects on IL-6 and IL-1β, crocin did not significantly modulate TNF-α levels, suggesting that its mechanism of action on this particular cytokine may involve different pathways.

Overall, these findings suggest that crocin exerts its anti-inflammatory effects primarily through the modulation of specific pro-inflammatory cytokines, such as IL-6 and IL-1β, rather than through broad-spectrum suppression of all inflammatory mediators. The differential effects of crocin on TNF-α compared to IL-6 and IL-1β observed in our study, as well as in others, highlight the need for further investigations to elucidate the precise molecular mechanisms underlying these selective actions.

Our findings align with previous studies demonstrating the anti-inflammatory effects of crocin and nobiletin in various disease models. As highlighted, crocin can suppress pro-inflammatory cytokine secretion and reduce ROS production by inhibiting NF-κBp65 translocation to the cell nucleus and regulating the NF-κB pathway. The modulation of the PI3K/Akt signaling pathway also emerges as a promising therapeutic target for crocin, contributing to the inhibition of the NF-κB pathway and, consequently, exerting anti-inflammatory effects (11, 28). In our DED model, crocin effectively reduced inflammation, potentially by targeting these pathways.

Furthermore, literature indicates that nobiletin can alleviate neuroinflammation and memory deficits in an LPS-induced mouse model by suppressing microglial activation and the secretion of pro-inflammatory cytokines such as IL-1β, TNF-α, iNOS, and COX-2 (29). Nobiletin achieved these effects by modulating the NF-κB, PI3K/AKT, and MAPK signaling pathways in microglial cells (30). Interestingly, in our study, crocin exhibited stronger anti-inflammatory effects compared to nobiletin, which could be attributed to differences in their active compounds or molecular mechanisms. Further investigations are warranted to elucidate the precise mechanisms underlying the differential effects of these natural compounds on TNF-α and other pro-inflammatory mediators in the context of DED (11, 30, 31).

Fluorescein staining, a widely used technique to assess corneal epithelial defects and ocular surface integrity, further corroborated the histological findings. The untreated DED group showed extensive corneal epithelial damage and increased fluorescein uptake, consistent with the pathophysiology of DED, where tear film instability and chronic inflammation contribute to the breakdown of the corneal epithelial barrier. In contrast, the betamethasone, crocin, and nobiletin treatment groups displayed reduced fluorescein staining, suggesting their protective effects on the corneal epithelium and their potential to mitigate ocular surface damage associated with DED. The ability of these compounds to preserve epithelial integrity is particularly noteworthy, as a compromised epithelial barrier can exacerbate inflammatory processes, perpetuating the vicious cycle of DED.

The observed beneficial effects of crocin and nobiletin in this study can be attributed to their well-documented anti-inflammatory and antioxidant properties (11). Crocin, in particular, exhibits potent antioxidant activities, which may contribute to its protective effects against oxidative stress-induced ocular surface damage in DED (32). Similarly, possesses strong anti-inflammatory and antioxidant features that likely contribute to its protective effects in DED. Nobiletin's antioxidant properties may also play a role in mitigating oxidative stress-induced damage to the ocular surface, a key factor in the pathogenesis of DED (33).

While the results of this study are promising, it is important to acknowledge certain limitations and suggest directions for future research. First, although the mouse model used is widely accepted for investigating DED, it may not fully replicate the complex pathophysiology of human DED. Human clinical trials are necessary to confirm the safety and efficacy of these compounds. Second, the specific mechanisms underlying the anti-inflammatory and epithelial-preserving effects of crocin and nobiletin in DED require further exploration. Future studies focused on molecular pathways and cellular targets could provide valuable insights into their mechanisms of action.

### 5.1. Conclusions

A significant limitation of our study is the lack of measurement of absorption and the concentration of these compounds in ocular tissues. This prevents a complete understanding of the bioavailability and pharmacokinetics of crocin and nobiletin in the eye. Future research should incorporate techniques such as high-performance liquid chromatography (HPLC) or mass spectrometry to quantify the concentrations of these compounds in various ocular structures over time, providing crucial information on their ability to reach target tissues and maintain therapeutic levels.

Additionally, our study focused on the individual effects of crocin and nobiletin without exploring potential synergistic or additive effects. We did not investigate how these compounds might interact when administered together, nor did we examine their combined effects with established drugs used in DED treatment. Future studies should consider evaluating combination therapies to determine whether crocin and nobiletin could enhance the efficacy of current standard treatments or exhibit synergistic effects when used together.

Another important aspect that warrants further investigation is the potential side effects of crocin and nobiletin. Although these compounds are naturally derived, they may still have adverse effects, particularly with long-term use. Toxicology studies and assessments of potential drug interactions are essential. Furthermore, research on bioavailability and ocular penetration following various administration routes is necessary to optimize drug delivery for therapeutic use. The relatively short duration of our study is another limitation, as it may not capture long-term effects. Additionally, the study focused on specific inflammatory markers, which may not fully represent the complexity of DED pathogenesis. Translating these findings to human use presents challenges, including differences in ocular anatomy and physiology between mice and humans, as well as potential issues with formulating these compounds for human application.

Despite these limitations, our research provides compelling evidence for the therapeutic potential of crocin and nobiletin in DED management. These natural compounds offer promising avenues for developing safer and potentially more effective treatments, which could improve the quality of life for millions affected by DED worldwide. Addressing these limitations in future studies would significantly enhance our understanding of crocin and nobiletin as potential therapeutic agents for DED and pave the way for more targeted and effective treatments.

## Data Availability

The dataset presented in the study is available on request from the corresponding author during submission or after publication.

## References

[1] Uchino M, Schaumberg DA (2013). Dry Eye Disease: Impact on Quality of Life and Vision.. Curr Ophthalmol Rep..

[2] Lemp MA (2008). Management of dry eye disease.. Am J Manag Care..

[3] Bron AJ, Tomlinson A, Foulks GN, Pepose JS, Baudouin C, Geerling G (2014). Rethinking dry eye disease: a perspective on clinical implications.. Ocul Surf..

[4] Marshall LL, Roach JM (2016). Treatment of Dry Eye Disease.. Consult Pharm..

[5] Wei Y, Asbell PA (2014). The core mechanism of dry eye disease is inflammation.. Eye Contact Lens..

[6] Bu J, Liu Y, Zhang R, Lin S, Zhuang J, Sun L (2024). Potential New Target for Dry Eye Disease-Oxidative Stress.. Antioxidants (Basel)..

[7] Ganesalingam K, Ismail S, Sherwin T, Craig JP (2019). Molecular evidence for the role of inflammation in dry eye disease.. Clin Exp Optom..

[8] Shoari A, Kanavi MR, Rasaee MJ (2021). Inhibition of matrix metalloproteinase-9 for the treatment of dry eye syndrome; a review study.. Exp Eye Res..

[9] Soeda S, Ochiai T, Shimeno H, Saito H, Abe K, Tanaka H (2007). Pharmacological activities of crocin in saffron.. Journal of Natural Medicines..

[10] Bastani S, Vahedian V, Rashidi M, Mir A, Mirzaei S, Alipourfard I (2022). An evaluation on potential anti-oxidant and anti-inflammatory effects of Crocin.. Biomed Pharmacother..

[11] Hashemzaei M, Mamoulakis C, Tsarouhas K, Georgiadis G, Lazopoulos G, Tsatsakis A (2020). Crocin: A fighter against inflammation and pain.. Food Chem Toxicol..

[12] Pashirzad M, Shafiee M, Avan A, Ryzhikov M, Fiuji H, Bahreyni A (2019). Therapeutic potency of crocin in the treatment of inflammatory diseases: Current status and perspective.. J Cell Physiol..

[13] Huang H, Li L, Shi W, Liu H, Yang J, Yuan X (2016). The Multifunctional Effects of Nobiletin and Its Metabolites In Vivo and In Vitro.. Evid Based Complement Alternat Med..

[14] Singh AP, Kandpal JB, Sharma RK, Chitme H (2021). Nobiletin a Biologically Active Phytoconstituent: Systematic Review.. Journal of Biologically Active Products from Nature..

[15] Shariat Razavi SM, Mahmoudzadeh Vaziri R, Karimi G, Arabzadeh S, Keyvani V, Behravan J (2020). Crocin Increases Gastric Cancer Cells' Sensitivity to Doxorubicin.. Asian Pac J Cancer Prev..

[16] Lien LM, Wang MJ, Chen RJ, Chiu HC, Wu JL, Shen MY (2016). Nobiletin, a Polymethoxylated Flavone, Inhibits Glioma Cell Growth and Migration via Arresting Cell Cycle and Suppressing MAPK and Akt Pathways.. Phytother Res..

[17] Sun J, Xu XM, Ni CZ, Zhang H, Li XY, Zhang CL (2011). Crocin inhibits proliferation and nucleic acid synthesis and induces apoptosis in the human tongue squamous cell carcinoma cell line Tca8113.. Asian Pac J Cancer Prev..

[18] Wilhelmus KR (2001). The Draize eye test.. Surv Ophthalmol..

[19] Yousefi-Manesh H, Aghamollaei H, Dehpour AR, Sheibani M, Tavangar SM, Bagheri M (2022). The role of saffron in improvement of ocular surface disease in a mouse model of Lacrimal Gland Excision-induced dry eye disease.. Exp Eye Res..

[20] Fakih D, Zhao Z, Nicolle P, Reboussin E, Joubert F, Luzu J (2019). Chronic dry eye induced corneal hypersensitivity, neuroinflammatory responses, and synaptic plasticity in the mouse trigeminal brainstem.. J Neuroinflammation..

[21] Lemp MA (2008). Advances in understanding and managing dry eye disease.. Am J Ophthalmol..

[22] Perez VL, Stern ME, Pflugfelder SC (2020). Inflammatory basis for dry eye disease flares.. Exp Eye Res..

[23] Solomon A, Dursun D, Liu Z, Xie Y, Macri A, Pflugfelder SC (2001). Pro- and anti-inflammatory forms of interleukin-1 in the tear fluid and conjunctiva of patients with dry-eye disease.. Invest Ophthalmol Vis Sci..

[24] Xu GL, Li G, Ma HP, Zhong H, Liu F, Ao GZ (2009). Preventive effect of crocin in inflamed animals and in LPS-challenged RAW 264.7 cells.. J Agric Food Chem..

[25] Fabiano A, De Leo M, Cerri L, Piras AM, Braca A, Zambito Y (2022). Saffron extract self-assembled nanoparticles to prolong the precorneal residence of crocin.. Journal of Drug Delivery Science and Technology..

[26] Hessen M, Akpek EK (2014). Dry eye: an inflammatory ocular disease.. J Ophthalmic Vis Res..

[27] Xiao Q, Xiong Z, Yu C, Zhou J, Shen Q, Wang L (2019). Antidepressant activity of crocin-I is associated with amelioration of neuroinflammation and attenuates oxidative damage induced by corticosterone in mice.. Physiol Behav..

[28] Xie Y, He Q, Chen H, Lin Z, Xu Y, Yang C (2019). Crocin ameliorates chronic obstructive pulmonary disease-induced depression via PI3K/Akt mediated suppression of inflammation.. Eur J Pharmacol..

[29] Cui Y, Wu J, Jung SC, Park DB, Maeng YH, Hong JY (2010). Anti-neuroinflammatory activity of nobiletin on suppression of microglial activation.. Biol Pharm Bull..

[30] Qi G, Mi Y, Fan R, Li R, Liu Z, Liu X (2019). Nobiletin Protects against Systemic Inflammation-Stimulated Memory Impairment via MAPK and NF-kappaB Signaling Pathways.. J Agric Food Chem..

[31] Gao J, Zhao F, Yi S, Li S, Zhu A, Tang Y (2022). Protective role of crocin against sepsis-induced injury in the liver, kidney and lungs via inhibition of p38 MAPK/NF-kappaB and Bax/Bcl-2 signalling pathways.. Pharm Biol..

[32] Sapanidou V, Taitzoglou I, Tsakmakidis I, Kourtzelis I, Fletouris D, Theodoridis A (2015). Antioxidant effect of crocin on bovine sperm quality and in vitro fertilization.. Theriogenology..

[33] Zhang L, Zhang X, Zhang C, Bai X, Zhang J, Zhao X (2016). Nobiletin promotes antioxidant and anti-inflammatory responses and elicits protection against ischemic stroke in vivo.. Brain Res..

